# Relationship Between Internet Use and Change in Health Status: Panel Study of Young Adults

**DOI:** 10.2196/22051

**Published:** 2021-01-13

**Authors:** Amanda Hunsaker, Eszter Hargittai, Marina Micheli

**Affiliations:** 1 Department of Communication and Media Research University of Zurich Zurich Switzerland; 2 European Commission Varese Italy

**Keywords:** internet use, health-related internet use, health status, panel data, young adults, internet, healthy living

## Abstract

**Background:**

Using the internet for health information is a widespread phenomenon documented in considerable scholarship. Less common, however, is the analysis of panel data to examine how internet use may relate to change in health status over time.

**Objective:**

This study examines whether internet use and internet use related to health are associated with a change in health status among young adults.

**Methods:**

We used a unique panel survey data set collected about young adults’ internet use in 2012 and 2016 (n=384). We applied logistic regression to examine the relationships between sociodemographics, internet experiences, frequency of health-related internet use, and sharing health content online with change in health status over time. We additionally examined the variables characterizing sharing health content online (via Facebook, Twitter, and email) in separate models.

**Results:**

In the second wave, over half (236/384, 61.5%) of the sample used the internet for health at least weekly. Approximately one-third (141/384, 36.7%) used Facebook for health-content sharing, while using Twitter and email for sharing health content were far less frequent (14/384, 3.6%, and 55/384, 14.3%, respectively). A change in health status occurred for 43.0% (165/384) of the sample; 18.5% (71/384) reported an improvement while 24.5% (94/384) reported a decline. Greater frequency of internet use was associated with health decline over time (*B*=–0.58, *P*=.02). We also found that frequent health-related internet use was related to enhanced health or maintained health (*B*=0.58, *P*=.03). Sharing health content on social media or email, however, was not related to young adults’ health changes.

**Conclusions:**

Young adults exhibit a pattern of using the internet for health that influences their health status. Our finding that frequent health-related internet use may promote improved or maintained health suggests that this type of online activity might also support healthy living.

## Introduction

The internet has become an important means through which people engage with health content; among US adults, well over half turn to the internet as a source of health information [1-3]. Young adults—individuals in their late teens and twenties [4]—report even higher levels of internet use for health [5-7]. Young adults are over 2 times as likely to search for a health provider online and over 3 times as likely to search for health information as their older counterparts even when controlling for other sociodemographic characteristics [8]. Younger age is continually reported to be an indicator of a higher likelihood of using the internet for health-related reasons [9,10].

A substantial body of work has documented the relationship between internet use and social inequalities [11-14]. However, how internet use relates to the production of health inequalities or differences in health conditions and care due to social factors is less known [15]. Internet researchers continue to grapple with whether and how the proliferation of digital technology benefits or harms the well-being of individuals and society [16].

The internet offers several pathways to seek and share health-related information that may enhance medical knowledge. Examining the relationship between internet use and health may therefore inform whether the internet has the potential to influence the state of health in positive or negative ways. We address this question by analyzing unique panel data about a diverse group of young adults to see whether their internet uses relate to a change in their health status over time. Panel data are especially relevant to such questions as they sidestep the limitation of cross-sectional studies, which do not allow for conjectures about whether health status is a result of or a cause for the way people use the internet.

Most studies explore health-related internet use in the form of health information–seeking: purposeful searching for health information using reliable sources or those of unknown reliability for one’s self or others [17,18]. Such use among young adults is strikingly high; 94% report looking for health information online while 76% have viewed or read about another individual’s health experiences [7]. Although such internet use is prevalent, social inequalities persist. Mirroring findings across all age groups [19-22], among young adults, women and those with higher socioeconomic status are more likely to seek health information online than men and people from less privileged backgrounds [6,7,23-25]. Differing from reports on older age groups [22], studies of young adults report little variation across racial and ethnic groups with regard to health information–seeking online [6,7,24].

Compared with the considerable literature that has looked at how people seek health content online, fewer studies have examined the sharing of such content [26]. Similar to research on content-sharing more generally [27], studies vary widely in how they conceptualize and measure the domains of shared health content. Studies also differ by whether they specify social media platforms. Facebook comprises the bulk of research describing posting behavior [28-30], but research has also considered other platforms such as Twitter [31] and health-focused online communities [32]. While some studies do use secondary data sets that leave platform unspecified [10,33], survey questions may group sharing health content with reading health content under the overarching domain of user-generated and shared content [8], which makes it difficult to know what to conclude about sharing in particular in such contexts.

Social network sites attract growing numbers of the US population for reading or sharing about health-related topics, including managing health concerns [1,3]. This figure is higher among young adults, with half sharing such content in 2016 [34]. Age group comparisons over time also find that younger individuals share health-related content more often than older and middle-aged adults [10]. Being younger and female as well as having lower income and education all associate with using social network sites for health [3,8,10,35]. Overall, we conclude that social inequalities related to health information–seeking online also appear to be associated with sharing health content online. These findings, as well as the wealth of research documenting the link between social inequalities and health outcomes [36], set the stage for examining the relationship between health-related internet use and health outcomes.

Research exploring the relationship between internet use and self-reported general health status finds varying results. Some studies point to a positive association where better health relates to both general internet use [29,37,38] as well as more frequent use of the internet [39-41]. The relationship between health-related internet use specifically and health status is mixed and, adding further uncertainty, this variation in relationships occurs whether modeling health status as an independent variable [20,21,42-45] or outcome variable [41,46-48]. We review these streams of research in turn below.

Most research treats health status as an independent variable and models health information–seeking as the outcome of interest. Studies analyzing cross-sectional data from the Pew Research Center found that poor health was associated with health-related internet use [21,43]. Worse health was also related to a greater number of health topics sought online [21]. Among adults who looked for health information online, individuals reporting poorer health searched for health content online more frequently and completed specific searches for health care and treatment [43].

However, studies have also related better health to health information–seeking online. Cotten and Gupta [20] found that those who were healthier were more likely to seek health information online than via offline sources. The same significant relationship between better health and health-related internet use occurred in a multinational sample of adults, although certain indicators of worse health (eg, long-term illness and disability, health care use) did link to more health-related internet use [42]. A secondary analysis found that while health status was not associated with health information–seeking online, presence of a health condition did have a positive relationship [44]. The above findings reveal a clear variation across research studies, warranting further inquiry.

Far fewer studies examine health status as the outcome of interest relative to health-related internet use, yet all report no significant direct relationship between health-related internet use and health state [41,46-48]. Most such studies use cross-sectional samples [47,48], while panel data are much less common [41,46]. Research that looks at other health outcomes such as the presence of health-promoting behaviors does report significant associations with health information–seeking online [49].

Similar to health outcomes associated with health-related internet use, just a handful of studies specifically examine the relationship between health-related social network site use and health status, although none of these studies model health as an outcome variable [3,10,35,40]. Findings are mixed; one study found a positive relationship between having a primary care provider and social network site use for health [35]. Other studies reported no association between health status and health-related social network site use [3,10].

The potential benefits of social media for health communication include a range of positive implications for individuals in terms of social-emotional support, greater and more tailored health content, and increased social interaction [50]. That said, the frequency of time spent online may have a detrimental effect on well-being. A systematic review examining the impact of social media on well-being among adolescents reported overall mixed findings but noted that greater time spent online related to increased risk of exposure to harm [51]. Examining whether and by what mechanisms internet use influences health therefore becomes all the more important to tease out, especially for a group that spends considerable time online—young adults.

The purpose of this study is to examine the relationship between internet use and health change. We use panel data, which to our knowledge is used less often, to explore whether general internet use and internet use related to health are associated with a change in health status. We examine this relationship within a sample of young adults as this population is of particular relevance due to their high consumption of online health information [5,7]. We define the domains of health-related internet use as the frequency of internet use for health and sharing health content online, offering a more in-depth description of a little-explored area than most existing studies.

## Methods

### Data Collection

We used a unique panel survey data set collected about young adults’ internet use in 2012 and 2016. The study received approval from the institutional review board of the principal investigator’s home university (Northwestern University) at the time of data collection.

We surveyed students from a required first-year writing course at a US midwestern public university, thus avoiding any bias associated with who may be more likely to take specific classes. We administered the survey to students in 86 of the 92 course sections that agreed to participate in the study. Of the students enrolled in the class, 80.5% (1325/1646) completed the questionnaire. Of these respondents, we focused our study on first-year students under age 30 years; these 1115 people make up our initial 2009 sample. We then administered waves 2 and 3 through postal mail. Wave 2 (2012) occurred 3 years after the initial data collection, and respondents were offered a US $25 gift card for their participation. Wave 3 (2016) followed 4 years later with a US $40 gift card for those who returned their survey. In both years, we sent an introductory letter before the survey and then followed up after the survey with 2 postcard reminders and one more copy of the survey. In wave 2, the response rate was 51.2% (571/1115), but we discarded 24 cases (24/571, 4.2%) for not passing the attention check question, resulting in 547 valid responses. Wave 3 achieved a 34.44% (384/1115) response rate in comparison to the baseline sample, while 70.20% (384/547) of respondents completing the 2012 survey also completed the 2016 survey. Those completing the survey in waves 2 and 3 comprise our sample, and they mirror wave 1 except for a somewhat smaller proportion of African Americans. We used measures of internet use from 2016 (wave 3) data. We used measures of health status from 2012 and 2016 to capture change in health over 4 years. Sociodemographics come from the 2009 (baseline) survey.

### Measures

#### Demographic Characteristics

We measured gender by asking participants whether they identify as male or female (see Multimedia Appendix 1 for all questions used in analyses). We collected additional data on age, race, and ethnicity separately. As a proxy for socioeconomic status, we asked respondents for the educational attainment of both their mother and father. We included the highest level of education completed by either parent in the analyses and recoded categories to high school or less, some college, and college degree or more.

#### Internet Experiences

As summarized in Table 1, we measured internet experiences in several ways: years of internet use, autonomy of use, frequency of use, and internet skills. We used data collected in 2016 for internet experiences with the exception of length of internet use which we measured in 2009. Measures of length of internet use were not repeated because years reported would increase proportionally in waves 2 and 3. We measured this by asking respondents how long they had been online. We measured weekly time spent online using categorical response options asking separately about weekdays and weekends, which we then recoded by multiplying the weekday response by 5 and the weekend response by 2, and then summing these for a possible range of 0 to 42. We logged this final number of hours to account for diminishing returns of additional time online in the regression models. To measure number of access locations, or autonomy of use, we asked respondents whether they use the internet from 10 different locations. As with number of hours online, we logged number of access points for logistic regression to address the diminishing returns of having greater numbers of internet access points. To gauge internet skills, participants completed a series of questions regarding their understanding of 27 internet-related terms [52]. Past research validated this measure with observational measures and used it with young adult samples [12,53].

**Table 1:** Sample characteristics (n=384).

Characteristics		Values
**Gender, n (%)**		
	Women	231 (60.2)
	Men	153 (39.8)
**Race/ethnicity, n (%)**		
	White	167 (43.5)
	African American	29 (7.6)
	Hispanic	85 (22.8)
	Asian	90 (23.4)
**Parental education, n (%)**		
	High school or less	94 (24.5)
	Some college	98 (25.5)
	College degree or more	188 (49.0)
	Missing	4 (1.0)
**Internet experiences, mean (SD)**		
	Years of use^a^ (in 2009)	5.6 (2.2)
	Number of access locations	7.8 (1.7)
	Weekly web hours	21.4 (10.5)
	Internet skills (1-5)	3.6 (0.8)

^a^Years of use: n=382.

#### Health-Related Internet Use

We assessed two domains of health-related internet use in wave 3 (2016): frequency of overall health-related internet use and sharing health content online. We measured frequency of using the internet to obtain health information using a single question with categorical response options. We dichotomized this variable for regression modeling to monthly or less and weekly or more. To measure sharing health content we asked about such activity in the past year for Facebook, Twitter, and email, separately. We treated the measures of the use of each platform as separate, dichotomous measures in regression modeling.

#### Health Status

In 2012 and 2016 we measured health status with an extensively used, single-item question derived from the 36-Item Short Form Health Survey [54]. This measure allowed for brevity in questioning and is significantly related to other measures of health, including health care use, functional status, varied health conditions, and mortality [55-57]. We determined change in health over time by taking the 2016 health status measure and subtracting it from the 2012 measure. Positive values indicated improvement, zero indicated no change, and negative values indicated health decline. We recoded this variable into a binary measure of whether someone’s health declined (=0) versus improved or stayed the same (=1).

### Analysis

Given that none of the correlations of our measures of internet experiences and health-related internet use were higher than 0.2 (i.e., there was no multicollinearity in our data set), we continued with regression modeling. We used logistic regression to examine the relationships between sociodemographics, internet experiences, frequency of health-related internet use, and sharing health content online with change in health status (level of significance: *P*<.05). We examined the variables characterizing sharing health content online (via Facebook, Twitter, and email) in separate models.

## Results

### Sociodemographics

Table 1 summarizes the sociodemographic characteristics of respondents who completed surveys in waves 2 and 3 of data collection. There were more women than men in the sample (231/384, 60.2% versus 153/384, 39.8%), and their proportion is almost identical to the first wave of the study. The vast majority of respondents were aged 18 or 19 years at the time of the first data collection, and aged 25 to 26 years at the time of wave 3. Since age does not vary in the sample, we do not include it in analyses. Less than half (167/384, 43.5%) were White, almost one-quarter (90/384, 23.4%) Asian, a slightly smaller number (85/384, 22.8%) identified as Hispanic or Latinx, and less than 1 in 10 (29/384, 7.6%) as African American. The remaining respondents (13/384, 3.4%) reported being Native American, other, or did not report race/ethnicity. We found considerable variation in parental education; almost half (188/384, 49.0%) had at least one parent who completed at least a college degree, while roughly one-quarter (98/384, 25.5%) had completed some college and a quarter (94/384, 24.5%) had no more than a high school degree.

### Internet Experiences

Descriptions of internet experiences are found in Table 1 and show that while most participants have considerable online experience, the standard deviations signal that there is plenty of variation in the sample.

### Health-Related Internet Use

Our results reveal both high prevalence and variation in internet use for health among young adults. Table 2 summarizes the frequency of such use. In 2016, over half (236/384, 61.5%) of the sample used the internet for health at least weekly, with percentages of the full sample roughly split between once a week, a few times a week, and daily. About a seventh (52/384, 13.5%) of the sample only did this a few times a year and just a handful (14/384, 3.6%) reported never using the internet for health. Sharing health content was a less common phenomenon. Facebook was the most common platform for health-content sharing with approximately one-third (141/384, 36.7%) of the sample doing so in 2016. Using Twitter and email for sharing health content were even less frequent.

**Table 2:** Characteristics of health-related internet use in 2016.

Characteristics		Values, n (%)
**Frequency of health-related internet use (n=384)**		
	Never	14 (3.6)
	Few times a year	52 (13.5)
	Monthly	82 (21.4)
	Weekly	85 (22.1)
	Few times a week	82 (21.4)
	Daily	69 (18.0)
**Sharing health content online (n=383)**		
	On Facebook	141 (36.7)
	On Twitter	14 (3.6)
	On email	55 (14.3)

### Change in Health Status

Table 3 describes the health status of the sample in 2012 and 2016. As expected for a young adult sample, in both years the majority of respondents reported either excellent or very good health. Although we found comparable descriptive findings across years, a notable number of young adults reported a differing health status from 2012 to 2016. A change in health status occurred for just under half (165/384, 43.0%) of the sample; about a fifth (71/384, 18.5%) reported an improvement (eg, fair to very good or very good to excellent) while a quarter (94/384, 24.5%) reported a decline (eg, excellent to very good or good to poor). Just over half (216/384, 56.3%) reported the same health status in 2012 and 2016. This is partly due to the fact that there is a ceiling effect. Over a third (136/384, 35.4%) of participants reported excellent health in 2012 meaning that these people could not have reported an improved health status in 2016 given our measure of health status. In fact, a fifth (77/384, 20.1%) of the sample remained in excellent health at the second time point. Given the young cohort, we did not have issues of floor effects with people reporting poor health in 2012 who then could not report worse than poor health in 2016. Just 2 respondents reported poor health in 2012, and both reported good health in 2016.

**Table 3:** Descriptives for health status and health status change (n=384).

Health status	2012, n (%)	2016, n (%)
Excellent	136 (35.4)	117 (30.5)
Very good	157 (40.9)	159 (41.4)
Good	73 (19.0)	91 (23.7)
Fair	15 (3.9)	11 (2.9)
Poor	2 (0.5)	4 (1.0)
Missing	1 (0.3)	2 (0.5)

### Factors Related to Health Improving or Staying the Same

Using logistic regression, we modeled sociodemographic and internet-use factors related to health change, where we looked at health improving or staying the same compared with a decline in health (level of significance: *P*<.05). We completed modeling in which we first added sociodemographic characteristics (gender, race/ethnicity, parental education) to the model, and then internet experiences (autonomy of use, frequency of use, internet skills) followed by frequency of health-related internet use. Finally, we added the variables measuring sharing health content online through different platforms (Facebook, Twitter, and email) each in its own model. Table 4 presents these findings.

In the first model, we found no significant relationships between sociodemographics and health change. Turning to the second step, where we added the variables measuring internet experiences, being female emerged as significant and associated with positive health change/status quo health. Less time spent on the internet was significantly associated with health getting better or staying the same while holding sociodemographic factors constant. Additionally, having better internet skills significantly related to health improving or staying the same. Autonomy of use, or the number of access points for using the internet, was not statistically significant.

The third model included a dummy variable for regular health-related internet use (operationalized as weekly or more). None of the sociodemographics (gender, race/ethnicity, parental education) were significant in this model. Lower frequency of internet use continued to relate to improved or the same health status, while better internet skills no longer associated with better or status quo health. Autonomy of use stayed statistically nonsignificant in the model. Our newly added variable, health-related internet use at least weekly, related to health improving or staying the same when sociodemographics and internet experience variables were held constant.

**Table 4:** Logistic regression on health status improving or staying the same.

Variable			Model 1		Model 2		Model 3		Model 4			Model 5			Model 6	
			*B* ^a^	*P* value	*B*	*P* value	*B*	*P* value	*B*	*P* value	*B*		*P* value	*B*		*P* value
**Sociodemographics**																
	Gender, female		0.42	.09	0.58	.03	0.52	.06	0.52	.06	0.54		.05	0.50		.07
	**Race/ethnicity**															
		White	ref^b^	ref	ref	ref	ref	ref	ref	ref	ref		ref	ref		ref
		Hispanic	–0.06	.85	0.03	.92	–0.03	.93	–0.03	.94	–0.01		.99	–0.02		.95
		African American	–0.71	.11	–0.56	.22	–0.62	.18	–0.62	.18	–0.62		.19	–0.62		.18
		Asian	0.19	.56	0.24	.46	0.25	.46	0.25	.46	0.24		.47	0.25		.45
	**Parental education**															
		High school or less	ref	ref	ref	ref	ref	ref	ref	ref	ref		ref	ref		ref
		Some college	–0.12	.75	–0.16	.67	–0.24	.52	–0.23	.53	–0.24		.52	–0.23		.54
		College degree or more	–0.03	.93	–0.13	.72	–0.19	.58	–0.19	.59	–0.20		.57	–0.20		.57
**Internet experiences**																
	Number of access locations		—	—	0.43	.33	0.26	.56	0.26	.56	0.29		.51	0.26		.56
	Web use, hours per week		—	—	–0.58	.02	–0.62	.01	–0.62	.01	–0.63		.01	–0.62		.01
	Internet skills		—	—	0.36	.03	0.32	.06	0.32	.06	0.37		.03	0.32		.06
**Health-related internet use**																
	Health-related internet use weekly or more		—	—	—	—	0.58	.03	0.59	.03	0.63		.02	0.56		.04
	On Facebook		—	—	—	—	—	—	–0.05	.86	—		—	—		—
	On Twitter		—	—	—	—	—	—	—	—	–1.16		.06	—		—
	Through email		—	—	—	—	—	—	—	—	—		—	0.21		.60
	Constant		0.96	.01	0.49	.70	0.83	.51	0.83	.51	0.63		.62	0.83		.51

^a^B: unstandardized beta coefficient.

^b^ref: reference.

We next added sharing health content online using Facebook, Twitter, and email by separately creating three different models. As before, no sociodemographics (gender, race/ethnicity, and parental education) or autonomy of use associated with health change in any of the final modeling. However, less time spent online continued to relate to better or similarly good health as before, as did weekly use of the internet for health. Better internet skills again had a significant association with better or status quo health when modeled with using Twitter to share health information but had no association when sharing health content on Facebook or email. Said another way, when we included sharing health information via Twitter in modeling, having better internet skills significantly related to health improving or staying the same. Since only a tiny portion of respondents in the sample use Twitter for sharing health information, we hesitate to make too much of this finding. We found no significant association across these platforms for health-information sharing. We also ran these models without controlling for health-related internet use; sharing using the various means we examined remained nonsignificant (Table 5).

**Table 5:** Logistic regression on health status improving or staying the same modeling sharing health content online.

Variable			Model 1		Model 2		Model 3	
			*B* ^a^	*P* value	*B*	*P* value	*B*	*P* value
**Sociodemographics**								
	Gender, female		0.58	.03	0.60	.03	0.50	.07
	**Race/ethnicity**							
		White	ref^b^	ref	ref	ref	ref	ref
		Hispanic	0.03	.94	0.06	.88	–0.02	.95
		African American	–0.56	.22	–0.56	.23	–0.62	.18
		Asian	0.24	.47	0.24	.47	0.25	.45
	**Parental education**							
		High school or less	ref	ref	ref	ref	ref	ref
		Some college	–0.17	.65	–0.16	.66	–0.23	.54
		College degree or more	–0.13	.71	–0.13	.71	–0.20	.57
**Internet experiences**								
	Number of access locations		0.41	.34	0.47	.28	0.26	.56
	Web use, hours per week		–0.58	.02	–0.59	.01	–0.62	.01
	Internet skills		0.36	.03	0.41	.02	0.32	.06
	**Sharing health content online**							
		On Facebook	0.08	.77	—	—	—	—
		On Twitter	—	—	–1.00	.10	—	—
		Through email	—	—	—	—	0.21	.60
	Constant		0.49	.69	0.29	.82	0.83	.51

^a^B: Unstandardized beta coefficient.

^b^ref: reference.

## Discussion

### Principal Findings

In this section, we reflect on our findings, discuss the limitations of our study, and suggest avenues for future research. In sum, having a decline in health significantly associated with greater overall time spent online and also less time explicitly using the internet for health. Sharing health content using different social network sites (Facebook or Twitter) or email did not relate to health change. We found that better internet skills related to positive health change or no health change but became nonsignificant when we included health-related internet use variables in the modeling, with the exception of adding health content shared on Twitter.

We found few previous studies that examined health over time in relation to internet use or health information seeking online [37,41,46,59] and the current findings offer a more in-depth look at the relationship between varying types and intensities of internet use and health change. In measuring health change over 4 years, our conceptualization of health improvement included young adults who reported an improvement from poorer health as well as those reporting no change in health. Young adults with no health problems may be accessing information to maintain their currently healthy state [60,61] and yet still gain a positive health change or maintain their health state. Studies examining the relationship between internet use and health in cross-sectional data have found that individuals reporting medical problems and those in a healthy state use the internet for health-related reasons [62].

The finding that greater frequency of internet use is associated with health decline differs from past research, where more frequent internet use associated with better health [40], including in a longitudinal study [37]. Although we recommend the interpretation of a causal relationship with caution, from one perspective having a decline in health may free up time to be online, as one may have health conditions that make it difficult to engage in other activities. While this interpretation may concern different health conditions in varying ways, previous research reports no difference in the frequency of internet use in relation to differing illness types [63]. Alternatively, spending too much time online may be a detriment to health status. The frequency of varying types of online activities may also matter. For example, a survey of first-year college students found that more hours on the internet shopping, doing research, and playing games significantly associated with more depressive symptoms, while more hours spent in online communication associated with decreased symptoms of depression [64]. However, the internet has changed considerably since that study so more work in this domain is needed.

In this paper, we found a clear association between health-related internet use and health change. Our finding that frequent health-related internet use may promote improved or maintained health suggests that this type of online activity might also support healthy living. Recent qualitative and quantitative research supports the notion that these topics are of interest to young adults [60,61]. Our findings differ from previous longitudinal research reporting no association between health-related internet use and change in general health [46]. Cross-sectional studies using young adult samples have also reported varied findings for the relationship between health-related internet use and health status [23,61,65]. One study found a positive relationship between health-promoting behaviors and health-related internet use [23], while another found no relationship between health-related internet use and health status [61]. Further adding to the diversity of study findings for this age group, other research found that young adults who used the internet for health information also reported more adverse health conditions [65]. This variation across study findings may be due to the large variety of health-related activities in which young adults engage online. One study reported that those searching for general health information online reported better health, while individuals searching for disease-specific information reported poorer health [39]. We recommend that future research include more details about the kinds of health topics that young adults explore online in relation to health outcomes.

We found no relationship between three different ways of sharing health content online (using Facebook, Twitter, and email) and health change. The low percentages of respondents using these platforms for health matters may account for our null findings, especially in light of our relatively small sample size. Although recent research notes a significant uptick in health-related internet use among young adults, social network sites were the least used sources and also considered the least reliable [61]. This distrust may hinder how young adults in turn share health content as they may be reluctant to post what they or their peers deem questionable information. Alternatively, we may see an increase in sharing health content online, as increased health information–seeking may create a wealth of online lay experts who have an expressed interest in sharing their health knowledge.

We found a significant association between being female and better or maintained health. Past studies show that male college students may engage in more risky health behaviors [66], yet they may also report better health than their female counterparts [67]. Differing health measures may indeed better explicate the relationship between health and varying ways of using the internet for health reasons as they relate to gender. Internet skills have often been associated with more capital-enhancing uses of the internet [12]—that is, uses from which they may benefit.

Most respondents reported their health as excellent or very good at both time points. Comparing these figures to national survey data of students enrolled in colleges or universities in the fall of 2012 [68] and 2016 [69] suggests that our sample reported better health overall at both time points and more respondents reported excellent health. However, unlike those studies, ours did not focus on health and thus did not prime respondents to think about health, which may explain the differences. It is also important to note that, compared with other studies that investigate the relationship of internet use and health, our study examined both as naturally occurring and is absent of an intervention design. Therefore, noting an association between these domains is all the more difficult to detect.

Health care professionals should understand the roles that health-related internet use plays for young adults, particularly if it has the potential to impact health in positive ways. Our results point to the potential importance of online tools for health that are specifically designed for young adults. To date, reviews find high-quality intervention studies specifically for this age group to be limited [70], although there is a growing body of studies using social network sites as platforms for health-behavior interventions for all ages [71]. How to harness such engagement among young adults for positive health behavior change warrants further exploration.

### Limitations

Our findings should be considered with some care. We measured the natural occurrence of internet use and health through self-reports. There may be unobserved variables affecting our findings. Our small sample size, due to attrition typical of panel studies, as well as the low variation in health status and low prevalence of sharing health content online require our findings to be interpreted with caution. Additionally, because our study began when the internet did not offer the multitudes of options it does today, our measures of its uses for health purposes are not as detailed as current options would warrant. Future studies with larger samples sizes and greater variation across measures may further explore whether, in fact, sharing health content online relates to health outcomes. Despite these limitations, given that panel studies of internet use are rare, the paper nonetheless makes a unique contribution to understanding the longer-term consequences of internet use for health.

### Conclusions

Analyzing a unique panel survey of a diverse group of young adults, this paper contributes to research on digital media by examining how internet use relates to change in health status over time. We find a relationship between internet use and longitudinal change in health, with frequent health-related internet use related to enhanced health or maintained health. We find no relationship of sharing health content on social media or email with health status. Given the relatively small size of our sample due to attrition in panel studies, future research may revisit that question on larger samples. Other measures of health outcomes, including specific health conditions and behaviors, as well as health care use, may also tease out the impact of internet use on health. Young adults exhibit a pattern of using the internet for health that influences their health status. Finding the avenues of internet use that may be most beneficial to their health and well-being is important to serving this generation just entering adulthood, as well as generations that follow.

## Appendix

Multimedia Appendix 1 Survey questions.
