# Supplementary material for: Relationship Between Internet Use and Change in Health Status: Panel Study of Young Adults
Source: J Med Internet Res. 2021 Jan 13;23(1):e22051. doi: 10.2196/22051 (PMC7840280; doi:10.2196/22051)
Supplement: Multimedia Appendix 1 [file jmir_v23i1e22051_app1.docx]

1. Appendix to Hunsaker, Hargittai & Micheli, JMIR 2020

Questions included in the paper are presented here in the order they are discussed in the paper.

# Demographic Characteristics (Wave 1, 2009)

Are you: ❑ Male ❑ Female

Are you Hispanic or of Latino origin?

❑ Yes
❑ No

What is your race? *Check all that apply.*

❑ White/Anglo/Caucasian/Middle Eastern

❑ Black/African American

❑ Asian, please specify: ______________________

❑ American Indian or Alaskan Native

❑ Other, please specify: ______________________

What is the highest level of education your father obtained?

❑ Less than high school degree

❑ High school degree

❑ Some college

❑ College graduate (for example: B.A., B.S., B.S.E)

❑ Advanced graduate (for example: master’s, professional, J.D., M.B.A, Ph.D., M.D., Ed.D.)

What is the highest level of education your mother obtained?

❑ Less than high school degree

❑ High school degree

❑ Some college

❑ College graduate (for example: B.A., B.S., B.S.E)

❑ Advanced graduate (for example: master’s, professional, J.D., M.B.A, Ph.D., M.D., Ed.D.)

# Internet Experiences (Wave 3, 2016, with exception of q. 6, which was asked in 2009)

When did you start using the Internet regularly (at least once a week)?

❑ In college

❑ Senior year of high school

❑ In high school, before senior year

❑ In middle school

❑ In elementary school or earlier

At which of these locations do you have access to the Internet, that is, if you wanted to you could use the Internet at which of these locations? *Check all that apply.*

❑ Your home

❑ Library or computer lab

❑ School (other than library or lab)

❑ Work

❑ Friend’s home

❑ Family member’s home

❑ Coffee house/Internet café

❑ Community center

❑ Outside (using wireless)

❑ On the go (using your cell phone, PDA, tablet, iPad, or roaming wireless)

On an average weekday, *not* counting time spent on email, chat and phone calls, about how many hours do you spend visiting Web sites?

🔾 None

🔾 More than zero, but less than 1 hour per day

🔾 1 hour

🔾 2 hours

🔾 3 hours

🔾 4 hours

🔾 5 hours

🔾 6 hours or more

On an average Saturday or Sunday, *not* counting time spent on email, chat and phone calls, about how many hours do you spend visiting Web sites?

🔾 None

🔾 More than zero, but less than 1 hour per day

🔾 1 hour

🔾 2 hours

🔾 3 hours

🔾 4 hours

🔾 5 hours

🔾 6 hours or more

How familiar are you with the following computer and Internet-related items? Please choose a number between 1 and 5 where 1 represents “no understanding” and 5 represents “full understanding” of the item.

|  | **None** | **Little** | **Some** | **Good** | **Full** |
| --- | --- | --- | --- | --- | --- |
| JPEG | 1 | 2 | 3 | 4 | 5 |
| Frames | 1 | 2 | 3 | 4 | 5 |
| Preference settings | 1 | 2 | 3 | 4 | 5 |
| Newsgroups | 1 | 2 | 3 | 4 | 5 |
| PDF | 1 | 2 | 3 | 4 | 5 |
| Refresh/Reload | 1 | 2 | 3 | 4 | 5 |
| Advanced search | 1 | 2 | 3 | 4 | 5 |
| Weblog | 1 | 2 | 3 | 4 | 5 |
| Bookmark | 1 | 2 | 3 | 4 | 5 |
| Bookmarklet | 1 | 2 | 3 | 4 | 5 |
| Spyware | 1 | 2 | 3 | 4 | 5 |
| Bcc (on email) | 1 | 2 | 3 | 4 | 5 |
| Blog | 1 | 2 | 3 | 4 | 5 |

How familiar are you with the following computer and Internet-related items? Please choose a number between 1 and 5 where 1 represents “no understanding” and 5 represents “full understanding” of the item.

|  | **None** | **Little** | **Some** | **Good** | **Full** |
| --- | --- | --- | --- | --- | --- |
| Tagging | 1 | 2 | 3 | 4 | 5 |
| Tabbed browsing | 1 | 2 | 3 | 4 | 5 |
| RSS | 1 | 2 | 3 | 4 | 5 |
| Wiki | 1 | 2 | 3 | 4 | 5 |
| Malware | 1 | 2 | 3 | 4 | 5 |
| Social bookmarking | 1 | 2 | 3 | 4 | 5 |
| Podcasting | 1 | 2 | 3 | 4 | 5 |
| Phishing | 1 | 2 | 3 | 4 | 5 |
| Web feeds | 1 | 2 | 3 | 4 | 5 |
| Firewall | 1 | 2 | 3 | 4 | 5 |
| Cache | 1 | 2 | 3 | 4 | 5 |
| Widget | 1 | 2 | 3 | 4 | 5 |
| Favorites | 1 | 2 | 3 | 4 | 5 |
| Torrent | 1 | 2 | 3 | 4 | 5 |

# Health-related Internet Use (Wave 3, 2016)

How often, if ever, do you use the Internet or the Web for the following?

| How often? | Never | Few times a year | Monthly | Weekly | A few times a week | Daily |
| --- | --- | --- | --- | --- | --- | --- |
| Health, fitness | 1 | 2 | 3 | 4 | 5 | 6 |

Have you shared any of the following content in the past year? For each, please indicate if you have shared it (a) on Facebook, (b) on Twitter, (c) through email, (d) through another site/service, or whether you did not share such content at all. For each, check all that apply.

|  | Facebook | Twitter | Email | Other, specify | Did not share |
| --- | --- | --- | --- | --- | --- |
| Links to health and fitness content | 🞎 | 🞎 | 🞎 | 🞎  ________ | 🞎 |

# Health Status (Wave 2, 2012; Wave 3, 2016)

In general, how is your health?

🔾 Excellent

🔾 Very good

🔾 Good

🔾 Fair

🔾 Poor
